# Microsatellite markers reveal multiple origins for Italian weedy rice

**DOI:** 10.1002/ece3.848

**Published:** 2013-10-31

**Authors:** Annabelle Grimm, Silvia Fogliatto, Peter Nick, Aldo Ferrero, Francesco Vidotto

**Affiliations:** 1 Institute of Botany, Karlsruhe Institute of Technology (KIT)Kaiserstr. 2, Karlsruhe, D-76131, Germany; 2 Dipartimento di Scienze Agrarie, Forestali e Alimentari, Università degli Studi di TorinoVia Leonardo da Vinci 44, Grugliasco, Torino, 10095, Italy

**Keywords:** Awn, plant morphology, population genetics, SSRs, weedy rice

## Abstract

Weedy rice (*Oryza sativa* L.) is one of the major issues of rice cultivation worldwide. In Italy, it infests about 70% of the total rice area. Different Weedy Rice populations can be distinguished based on variable morphological and physiological traits; however, little is known about genetic differentiation and origin of Italian weedy rice populations. The objective of this study was to genetically and morphologically characterize and compare different Italian weedy rice populations selected on the basis of different phenotypes. The main Italian rice territory was divided into 10 geographical areas in which 40 weedy rice populations were collected and grouped according to the awn traits. All the individuals of the populations were morphologically characterized according to plant and seed traits. Genetic characterization was performed using 19 SSR markers on all the collected accessions, and several rice cultivars, including some very old (late 19th century), nowadays are no longer cultivated. ANOVA showed that morphological plant and seed traits were significantly affected by the collection area and awnedness group. The importance of the awn morphology was also reflected in the Bayesian clustering where, despite a relatively low genetic diversity, the clusters displayed different awn types. An UPGMA dendrogram confirmed the clusters detected in STRUCTURE analysis and also revealed a grouping of certain old cultivars with the weedy rice, suggesting a common origin.

## Introduction

Weedy rice (*Oryza sativa* L.) is one of the most troublesome weeds in rice cultivation worldwide, causing serious yield losses and affecting rice milling and seed trade (Gressel 2005). Its common name refers to the fact that it is comprised of types of the genus *Oryza*, which grow spontaneously and are morphologically similar to rice cultigens (Delouche et al. 2007). In Europe, particularly in Italy, these types since the early 20th century have been designated as *O. sativa* var. *sylvatica* or *O. sativa* f. *spontanea*, because they are difficult to distinguish from rice cultigens. However, in the meantime, it is clear that weedy rice is not a separate taxon and therefore should be termed as *O. sativa* (Jacometti 1912; Vidotto and Ferrero 2005).

Weedy rice differs from rice cultigens by morphological and physiological traits that are related to both vegetative and reproductive structures. In particular, weedy rice is characterized by high tillering ability, a tall habit, long and light green leaves, caryopses with red pericarp, variable hull coloration, and awnedness (Burgos et al. 2006; Shivrain et al. 2010; Fogliatto et al. 2011). Fast growth, efficient use of nutrients, resistance to drought, early grain shattering, and pronounced seed dormancy render weedy rice highly problematic (Valverde 2005; Delouche et al. 2007; Shivrain et al. 2010). Some of these traits, such as seed dormancy, shattering, and grain pigmentation had been lost in the cultigen during domestication and are central for adaptation and persistence of the weed in the agricultural environment (Harlan and deWet 1965; Gu et al. 2005).

The degree to which these domestication-associated traits are expressed varies considerable among different populations or ecotypes of weedy rice (Fogliatto et al. 2012). As most widespread subtypes of weedy rice, the “strawhull awnless” and the “blackhull awned” types can be defined by both morphology and genetic markers (Gealy et al. 2002). However, the diversity of types increases progressively, whereas in the 1850s four types of weedy rice were reported for North America (Craigmiles 1978), this number had increased to more than 50 different biotypes presently found in the same area (Noldin et al. 1999). The adaptation of weedy rice to different environments and cropping systems is correlated with a high biological diversity (Cao et al. 2006; Reagon et al. 2010). It is not clear to what extent this diversity is based on genetics or on developmental plasticity.

The exact origin of weedy rice and its high differentiation into highly diverse populations are far from being understood. Previous studies using SSR (Simple Sequence Repeats) and RAPD (Random Amplification of Polymorphic DNA) markers had suggested that some weedy rice populations are closely related to *indica*, but others to *japonica* rice cultigens (Gealy 2005). As suggested by recent studies, hybridization between weedy rice and cultigens but also between different accessions of weedy rice represents an important source for the diversity of weedy rice (Reagon et al. 2010). In addition, introgression, mutation, selection, genetic drift, and gene flow have all been proposed as driving forces for weedy rice origin and diversity (Kuroda et al. 2007).

Little is known about origin and genetic background of weedy rice in Italy. A recent study (Jiang et al. 2012) reported that some of the studied populations of Italian weedy rice were genetically similar and clustered separately from rice cultigens, whereas the remaining populations were closer to cultigens of rice. The same study suggested allele transfer between populations in combination with outcrossing and introgression from rice cultigens as main factors responsible for the variability in weedy rice. Although the exact start point for rice cultivation in Italy is not known, a relevant agricultural use can be traced back to the period between the 13th and 15th century (Spada et al. 2004; Ferrero and Vidotto 2010; Faivre-Rampant et al. 2011). Although weedy rice in Italian rice fields was already reported as early as the beginning of the 19th century (Biroli 1807), before 1960, weedy rice infestations were not causing major problems, because they were controlled by the practice of seedling transplanting and manual weeding. However, when direct seeding came into practice after 1960, infestations with weedy rice became progressively severe (Tarditi and Vercesi 1993; Vidotto and Ferrero 2005). This development became accelerated dramatically in Italy and all over Europe, when in the 1980s due to consumer demand, cultivation of *japonica* rice varieties with morphological traits similar to *indica* increased. These semidwarf varieties mimicking the *indica* ones are far less competitive relative to weedy rice as compared to the traditional varieties used before. Today, weedy rice is reported in 70% of European paddy fields, and continuous rice monocropping is considered to further boost its spread (Català et al. 2002).

Because recent studies have shown clustering of weedy rice and cultigens, we conducted a study to investigate whether cultigens might be involved in the emergence of weedy rice in northern Italy. The populations of weedy rice and cultigen samples were characterized and compared using 19 highly resolving SSR markers and morphological methods. The cultigens included not only cultivars used at present, but also those cultivated in Italy during the late 19th century, that is, the time when weedy rice became significant in Italy. The weedy rice accessions were systematically sampled over the core region of Italian rice cultivation. The comparison of genetics and morphology data suggests a correlation of a “weedy lifestyle” with awnedness. Although our study cannot fully uncover the origins of weedy rice in Italy, the genetic structure of the samples in combination with molecular phylogeny show that multiple events must have led to today's weedy rice pool in Italy. At least some of these events must have taken place in cultigens. Even though additional studies including other samples are required to fully unravel the history of weedy rice in Italy, this study sheds light on some of its origins.

## Materials and Methods

### Sampling of the biological material

**Figure 1:**
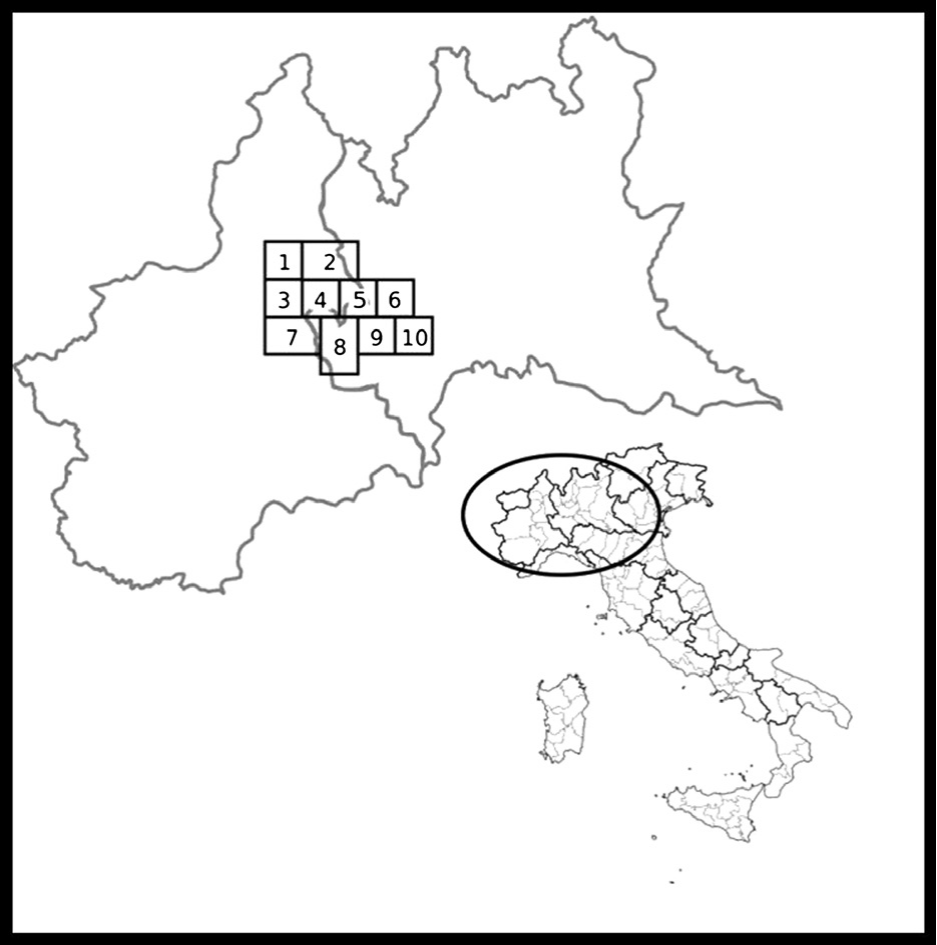
Collection area of the 40 weedy rice populations.

During 2009, a survey was carried out on about 130,000 hectares of rice fields in Northwest Italy. The territory was divided into ten areas with homogeneous cultivation practices; within each area, from three to five fields heavily infested with weedy rice were chosen (Fig. 1). The selected fields were georeferred by acquiring GPS coordinates. The weedy rice plants from each field were grouped based on morphological traits, such as plant height, node coloration, panicle habit, hull coloration, presence and length of awns. Each group was considered as a “population”. A total of 40 populations were identified. For each of these morphologically defined populations, a pooled sample of caryopses was obtained from ten individuals randomly selected.

### Morphological characterization

**Figure 2:**
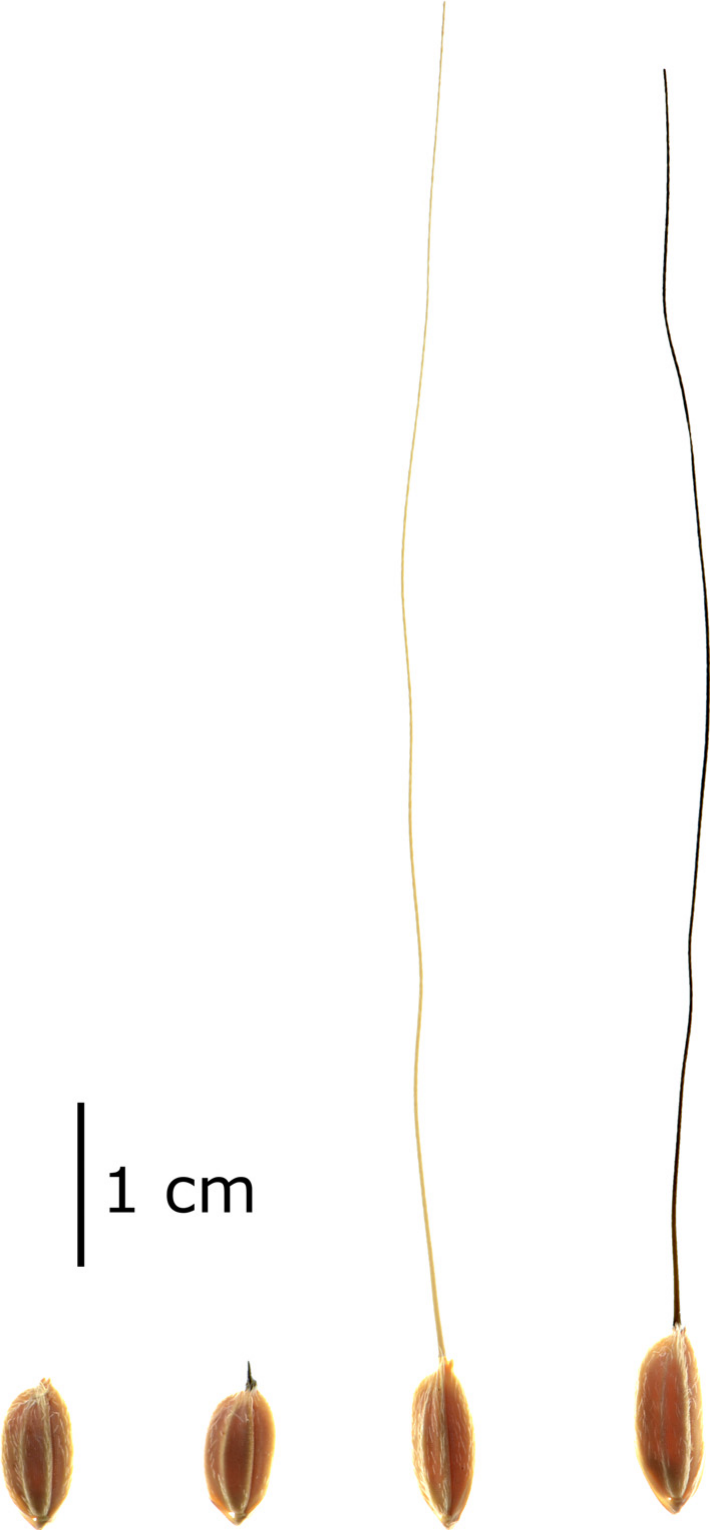
Seeds of weedy rice with different awnedness (from left to right: awnless, mucronate, and awned seeds, respectively).

The collected populations were characterized with respect to heritable morphology by sowing them side by side in asame field plot the subsequent year. Each population was seeded in 1 × 2 m plot, following a RCBD design, with three replications. From each population, 12 plants (four plants for each replication) were randomly sampled and characterized morphologically including vegetative and generative traits, with both continuous and categorical variables. When plants reached maturity, seeds from 3 to 4 individuals for each population were collected by hand for genetic analysis, yielding a total of 150 accessions. The morphological traits included in this study were as follows: plant height, number of seeds per panicle, panicle length, seed weight, anthocyanin coloration of nodes, panicle inclination in relation to stem, and hull coloration. The traits were assessed following the IRRI Standard Evaluation System for Rice (IRRI 2002) and the Protocol for Distinctness, Uniformity and Stability test for *O. sativa* provided by UPOV (2004). These traits were selected because they are either related to “weedy behavior”, or allow discrimination between weedy rice from cultigens or both. Based on the presence and pigmentation of the awn, four main groups were identified and termed awnless, mucronate (with an awn length shorter than 2 mm and/or having black coloration of the *apiculi*, Fig. 2), straw awned (awn straw colored, longer than 2 mm), and black awned (awn black colored, longer than 2 mm). To test statistically the influence of collection area and awn features on morphological traits, a MANOVA test was carried out. To test for differences in morphological traits depending on collection area and awn features, mean values were assayed using the REGWR test (Fig. 2).

### DNA extraction for genetic analysis

For genetic characterization, all 150 accessions of weedy rice corresponding to 40 populations, as well as 20 cultigens (including the varieties Flipper, Creso, Thaibonnet, Gladio, OriginarioCinese, Centauro, Ostiglia, Ranghino, Balilla, Carnaroli, Vialone Nano, Baldo, Sirio, Selenio, Prometeo, Artiglio, Bertone, Arborio, Loto, and Lady Wright) were included in the present study. The cultigens selected for this study are either commonly grown in Italy, or they had been common in the past. Three seeds from each weedy rice accession and cultigen were, respectively, sown on cell trays in the greenhouse, harvested at the 3-leaf stage, shock-frozen in liquid nitrogen, and stored at −80°C. Seedlings were ground using a high-throughput disruptor (TissueLyser, Qiagen, Hilden, Germany), and the DNA was extracted using the DNeasy Plant Mini Kit (Qiagen) as follows: after homogenization, 5 mL of preheated (65°C) extraction AP1 buffer, and 10 μL of RNase A were added to about 1 g of each sample and vortexed at high speed. The samples were incubated at 65°C for 10 min and mixed periodically by inverting the tube to lyze the cells. Then, 1.8 mL of AP2 buffer were added to each sample, followed by mixing and 10 min of incubation on ice. Next, samples were centrifuged at 5000 g (Eppendorf, 5417 R; Hamburg, Germany) for 5 min at 20°C, and the supernatant filtered through a QIAshredder Mini spin column (Qiagen) and placed in a collection tube and immediately spun at 5000 g for 5 min at 20°C. The lysate was transferred to a 2-mL tube without disturbing the pellet containing some cell debris. A mixture of 1.5 volumes of AP3 buffer and ethanol was added to the samples and mixed. The samples were transferred into a DNeasy Mini Spin Column in a reaction tube, centrifuged at 5000 g for 2 min, the flow-through discarded, and 12 mL of Buffer AW (wash buffer) added to the DNeasy Mini Spin Columns. After centrifugation for 2 min at 5000 g to dry the membrane, the flow-through was discarded, and the DNeasy Mini Spin Column with the processed sample transferred to a new 50-mL tube; 1 mL of AE buffer (elution buffer) was added to the column membrane and incubated at 20°C for 5 min. After centrifugation for 2 min at 5000 g for elution, further 750 μL of AE buffer were added and centrifuged again at the same speed. The two eluates were pooled and stored at −20°C.

### Microsatellite PCR

Nineteen microsatellite markers (SSRs) spread over the different rice chromosomes and tested in previous studies (Cao et al. 2006) were selected for this work. The corresponding oligonucleotide primers are indicated in Table 1. The PCR mixture (25 μL) contained 20 mmol/L TRIS, 50 mmol/L KCl, 3 mmol/L MgCl_2_, 0.25 mmol/L of dNTPs, 1 μmol/L of each oligonucleotide primer, 1 unit Taq polymerase (Taq DNA polymerase, Qiagen), and 3 μL of a 1:10 dilution of ca. 10 ng/μL genomic DNA. PCR was conducted after 3 min at 94°C in 40 cycles at 92°C for 1 min, annealing at 56°C for 1 min, synthesis at 72°C for 1.5 min, and a final cycle of 10 min at 72°C. Amplificates of different sizes and fluorescent labels were multiplexed and run on a capillary electrophoresis sequencer/genotyper, ABI 3730xl (Applied Biosystems, Monza, Italy). GeneMapper® Software, version 4.0 (Applied Biosystems) was used to identify the alleles, and the sizing was carried out with the internal size standard GS500LIZ (Applied Biosystems) containing 16 fragments with a size ranging from 35 to 500 bp (Chen et al. 2011). Amplification anomalies were detected for the three markers RM14, RM44, and RM55.

**Table 1:** Data of the SSR markers used

SSR	Chr	Fw 5′–3′	Rev 5′–3′	*N*_a_	*H*_e_	*H*_o_	bp range
RM 11	7	tctcctcttcccccgatc	atagcgggcgaggcttag	4	0.421	0.014	140–157
RM 14	1	ccgaggagaggagttcgac	gtgccaatttcctcgaaaaa	7	0.300	0.333	192–200
RM 17	12	tgccctgttattttcttctctc	ggtgatcctttcccatttca	2	0.482	0.007	175–202
RM 19	12	caaaaacagagcagatgac	ctcaagatggacgccaaga	2	0.014	0.000	231–262
RM 21	11	acagtattccgtaggcacgg	gctccatgagggtggtagag	6	0.332	0.007	148–175
RM 44	8	acgggcaatccgaacaacc	tcgggaaaacctaccctacc	4	0.360	0.269	124–128
RM 55	3	ccgtcgccgtagtagagaag	tcccggttattttaaggcg	4	0.506	0.241	248–252
RM 84	1	taagggtccatccacaagatg	tgcaaatgcagctagagtac	1	0.000	0.000	126
RM 167	11	gatccagcgtgaggaacacgt	agtccgaccacaaggtgcgttgtc	5	0.115	0.000	101–167
RM 180	7	ctacatcggcttaggtgtagcaacacg	acttgctctacttgtggtgagggactg	2	0.059	0.007	123–126
RM 211	2	ccgatctcatcaaccaactg	cttcacgaggatctcaaagg	1	0.000	0.000	158
RM 212	1	ccactttcagctactaccag	cacccatttgtctctcattatg	2	0.343	0.000	131–133
RM 215	9	caaaatggagcagcaagagc	tgagcacctccttctctgtag	4	0.462	0.000	163–171
RM 219	9	cgtcggatgatgtaaagcct	catatcggcattcgcctg	4	0.640	0.020	212–220
RM 230	8	gccagaccgtggatgttc	caccgcagtcacttttcaag	4	0.033	0.007	268–274
RM 253	6	tccttcaagagtgcaaaacc	gcattgtcatgtcgaagcc	4	0.595	0.000	148–153
RM 276	6	ctcaacgttgacacctcgtg	tcctccatcgagcagtatca	5	0.507	0.028	100–152
RM 280	4	acacgatccactttgcgc	tgtgtcttgagcagccagg	2	0.425	0.000	186–188
RM 289	5	ttccatggcacacaagcc	ctgtgcacgaacttccaaag	1	0.000	0.000	104
Average				3.368	0.295	0.049	

*N*_a_ number of alleles, *H*_*e*_, expected heterozygosity, *H*_*o*_, observed heterozygosity.

### Analysis of the SSR data

The raw SSR data were first analyzed using the freeware program Identity (http://www.uni-graz.at/∼sefck/) estimating allele number, and expected and observed heterozygosity (*H*_e_, *H*_o_). Genetic distances of the individual SSR allele lengths were calculated by the Microsat software (http://hpgl.stanford.edu/projects/microsat/) using the Chord distance (Cavalli-Sforza and Edwards 1967). The overall *F*_st_ values were calculated using the GENEPOP software, version 4.0.10 (Raymond and Rousset 1995). The software Structure 2.2 (Pritchard et al. 2000) was used to identify a model-based (Bayesian clustering) genetic structure in the SSR data. This program uses multilocus genotype data to investigate population structure by a Markov Chain Monte Carlo algorithm (MCMC), clustering individuals into K distinct populations by minimizing Hardy–Weinberg disequilibrium and linkage disequilibrium between loci within groups. The program was run at the default settings with an initial burnin period of 20,000 and 100,000 MCMC repeats. K was estimated from 2 to 10, and calculations for each K were repeated 20 times. The best fitting K was calculated by the Evanno method using the website program STRUCTURE HARVESTER (Earl and vonHoldt 2012).

### Phylogenetic tree

The phylogenetic tree was constructed using the MEGA 5.0 software (Tamura et al. 2011). A distance tree was calculated using the UPGMA algorithm on the genetic distances obtained from the SSR data of the 150 weedy rice and the 20 cultigen accessions. The representation in circle topology was chosen for better visualization for the number of accessions displayed in the tree.

## Results

### Morphological characterization

The collected weedy rice populations were in total 25% awnless, 20% mucronate, 25% straw awned, and 30% black awned. Area of collection and extent of awnedness could explain a significant part of the variation in the morphological plant and seed traits, as found in a MANOVA test, while the interaction between the two factors (area × awnedness group) was not significant (Table 2).

**Table 2:** Multivariate ANOVA of the effect of the area of collection and seed awnedness and their interaction on some morphological traits according to Pillai's Trace test (*P* ≤ 0.05)

Effect	Value	*F*	df	Error df	Significance *P* value
Intercept	0.9	8733.4	15.0	92.0	0.0
Area	2.3	2.2	135.0	900.0	0.0
Awnedness	1.8	10.0	45.0	282.0	0.0
Area × Awnedness	3.1	1.1	375.0	1590.0	0.9

In particular, weedy rice populations collected in the Eastern part of the sampled territory (areas 6 and 10, Fig. 1) consisted of taller plants with longer flag leaf and longer seeds (data not shown). This region differs from the rest of the sampled territory by sandy soils and significant differences in rice cultivation such as an increasing adoption of dry seeding and crop rotation.

**Figure 3:**
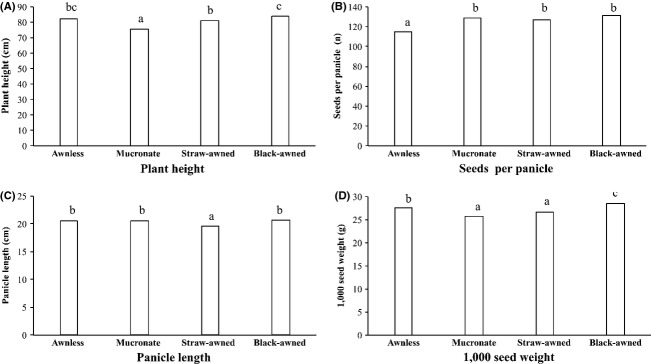
Plant (A and C) and seed traits (B and D) of weedy rice populations grouped according to awnedness (continuous variables). Values sharing the same letter are not significantly different according to Tukey's test (*P* ≤ 0.05). Comparisons were made among awnedness degree groups within each variable.

**Figure 4:**
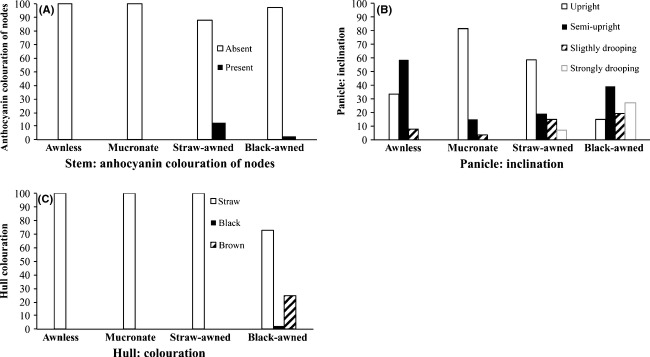
Plant (A and B) and seed traits (C) of weedy rice populations grouped according to awnedness (categorical variables).

When the weedy rice populations were grouped according to awn traits (Fig. 2), certain correlations with morphological traits could be detected (Figs 3, 4). Mucronates were significantly shorter, while black awned were significantly taller than the other populations. Straw awned populations showed a slightly, but significantly shorter panicle length, whereas all the other populations were characterized for having similar panicle length. The number of seeds per panicle was reduced by about 10% in the awnless compared with the other groups. This trait was inversely related to seed weight in awnless, mucronate, and straw awned populations. In particular, mucronate and straw awned populations produced lighter but more numerous seeds compared with awnless, which showed the opposite behavior. Conversely, black awned populations produced a high number of heavy seeds. Anthocyanin coloration of nodes was observed in the awned populations only (Fig. 4) reaching about 12% in the straw awned type. Similarly, the usual straw-color of the hull differed in the black awned populations, where about 24.5% and 2.4% of populations had brown and black hulls, respectively. The inclination of the panicle in relation to stem varied within each population. In general, the majority of populations showed upright (mucronate and straw awned groups) or semiupright (awnless) panicles. This trait was rather homogeneous in the mucronate group, as more than 80% of populations showed upright panicles. By contrast, the black awned group was the most diverse with all four manifestations of this trait (upright, semiupright, slightly drooping, and strongly drooping) representing with frequency values ranging from about 15–39%.

### Genetic characterization

One hundred and fifty individuals from 40 weedy rice populations and 20 cultigens were investigated with 19 SSR markers covering all rice chromosomes. A total of 64 alleles were found among the weedy samples with an average of 3.368 alleles per locus. The locus with the highest number of alleles was RM14 with 7 alleles (Table 1). Three of the 19 loci were not polymorphic (RM84, RM211, RM289). The mean *H*_e_ indicates a relatively high genetic diversity. The observed heterozygosity (*H*_o_), with an average of 0.049 is remarkably lower compared with *H*_e_. The pairwise F_st_ values for the populations ranged from 0 to 0.685 (data not shown) with no clear trend. Thus, the differentiation between the individual populations is highly variable, but the overall *F*_st_ for all 40 populations was 0.179, falling into the range between 0.15 and 0.25 that according to Wright (1978) indicates substantial differentiation. To test, whether the differentiation between the populations of weedy rice is linked with geographical distance, a spatial structure analysis as shown in Shivrain et al. (2010) was performed. This method compares genetic and geographical distance searching for correlation. The autocorrelation coefficient, *r*, obtained was 4 × 10^−5^, which argues against defined geographic origins for the spread of weedy rice.

**Figure 5:**
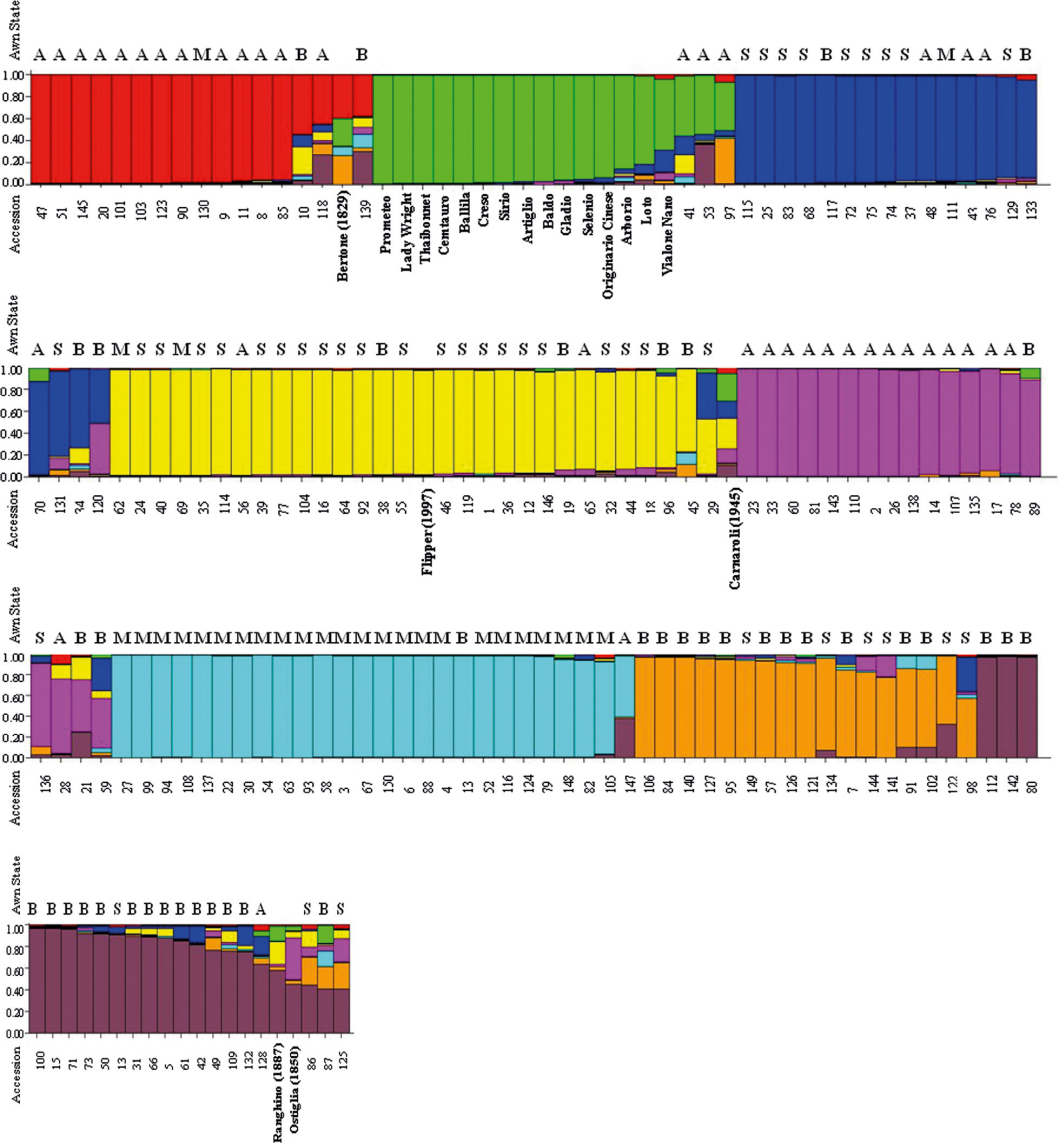
Plot of the results calculated with STRUCTURE. Different populations are shown in different colors. Each bar represents one individual and its affiliation to the single populations. The numbers of the accessions and the names of the cultivars are shown under the bars. The awn state for each individual is shown above the bar (A = Awnless, B = Black awned, M = Mucronate, S = Straw awned). In the case of cultivars clustering together with weedy rice, the year of its invention is given in brackets.

The population structure of the weedy rice accessions was assessed by Bayesian clustering using the software Structure 2.2 (Pritchard et al. 2000). The data set was best approximated by a model based on *K* = 8 populations defining eight major clusters (Fig. 5). When awn morphology of the individuals was integrated into these clusters, clear, but not exclusive correlations became evident: most cultigens grouped into a separate cluster distinct from the weedy rice clusters. However, four cultigens were located in different weedy rice clusters. Three of the four cultigens (Carnaroli, Ranghino, Ostiglia, and Bertone) were used in the 19th century, but are outdated in the meantime. Only one of these cultigens (Flipper) is currently used. It should be noted that the grouping by awn traits did not fully coincide with the genetically defined clusters. For instance, the majority of awnless accessions was grouped into either the red or the pink cluster, but was also to a small extent interspersed into the other clusters. Nevertheless, in all of the weedy rice clusters, one type of awn morphology clearly dominated, indicating a correlation between awn morphology and genetic distance.

**Figure 6:**
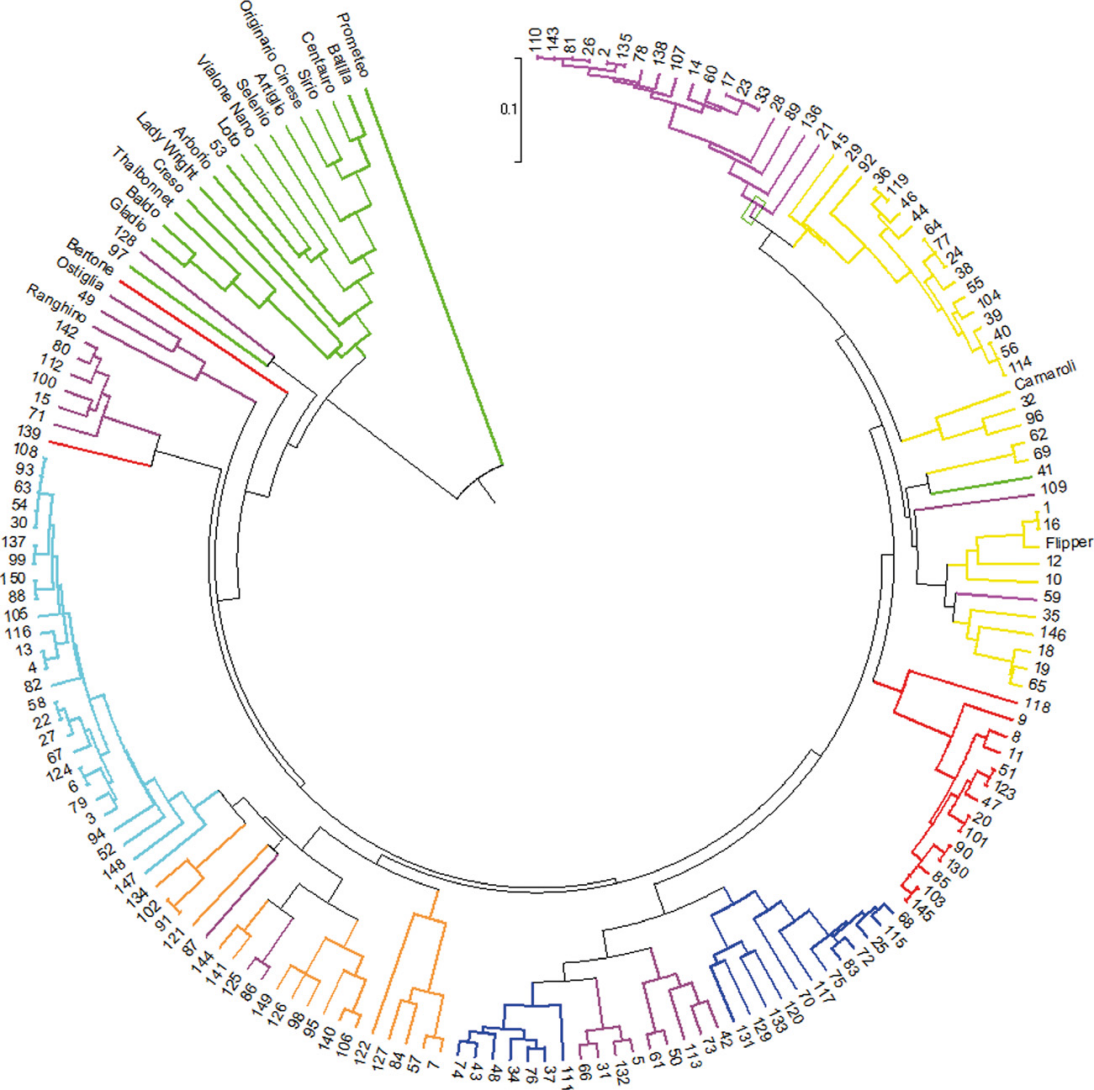
UPGMA dendrogram based on the genetic distance of the samples in this study obtained from the 19 SSR markers used. The affiliation to the population of the STRUCTURE analysis for each individual is represented in the same color code as used in Fig. 3. Weedy rice samples are labeled by their number used in the experiments, for the cultivars their common names are used. The dendrogram is shown in circle topology for better overview of the number of individuals represented.

To probe the relationship of weedy rice with the regional extant and outdated cultigens with higher resolution than in the Bayesian clustering, genetic distances were calculated over the entire set of weedy rice and cultigen accessions and a dendrogram constructed based on the UPGMA algorithm (Fig. 6). The affiliation to the Bayesian clusters was represented by the same color code. By the higher resolution, some of the clusters were split into different clades of the tree, but the general topology inferred from the Bayesian clustering was preserved to a high degree. Strikingly, the same five cultigens that had already been identified as outliers in the Bayesian clustering reappeared outside the cultigen clade inmidst of weedy rice accessions: the 19th century cultigens Carnaroli, Ranghino, Ostiglia, and Bertone, and the contemporary Flipper. In addition, the cultivar Prometeo behaved as outgroup with respect to the cultigen clade.

## Discussion

The origin of weedy rice in most regions of the world remains unclear, but is highly relevant to design sustainable strategies for the control of this weed. The great genetic variability found in this weed (Cao et al. 2006; Xia et al. 2011; Jiang et al. 2012) in combination with a long domestication history of the cultigen ranging back for more than 6000 years, makes it difficult or even impossible to reconstruct the evolution of weedy rice. The situation in Italy differs from other regions as, for instance, East Asia (Faivre-Rampant et al. 2011), because rice cultivation was adopted relatively recently. The breeding of Italian cultigens is even more recent, such that there exists a written documentation on the history of these cultigens. This specific situation provides a perfect model area to study the emergence and development of weedy rice.

This study was aimed to identify possible origins of weedy rice types found in the main Italian rice growing area by relating morphological and genetic diversity in a large set of weedy rice accessions as compared to a large set of cultigens. These cultigens not only comprised the cultivars currently used in the area under investigation, but also historical cultigens representing early stages in the generation of native Italian cultivars ranging back to the early 19th century.

We observed a great morphological diversity among the sampled weedy rice accessions, especially in relation to awn traits, where four groups (awnless, mucronate, black awned, and straw awned) could be detected. This was accompanied by a substantial, but not overwhelming, genetic variability both between morphological groups (as indicated by the values for *F*_st_), but also between the individual accessions (as indicated by the values for *H*_e_). Bayesian clustering revealed several sets of weedy rice indicating multiple origins of weedy rice. Whereas recent cultigens formed a separate cluster, four of the old cultivars clustered together with different sets of weedy rice and are therefore represent prime candidates for the ancestry of Italian weedy rice.

### Italian weedy rice: indications for a neophytic founder effect?

Although the genetic variability in the Weedy accessions as monitored by the absolute values for *F*_st_ and H_e_ for weedy rice appears substantial at first glance, a second look reveals that the observed heterozygosity (*H*_o_) was very low compared with the relatively high *H*_e_ values. Also the number of nonpolymorphic loci is lower as compared to results obtained with weedy rice in Northeastern China (Cao et al. 2006) were all loci were polymorphic using the same marker set. The fact that the same set of markers monitors a lower level of genetic diversity in weedy rice from a relatively young rice growing area as compared to an area with a much more ancient history points to a low genetic diversity in the ancestral population giving rise to the Italian Weedy accessions. This type of genetic bottleneck, known as Founder Effect, is often observed during neophytic invasions (Dlugosch and Parker 2008; Voss et al. 2012). The relatively high homozygosity and the occurrence of nonpolymorphic loci must be seen in context with the relatively small sampling area and the typical self-pollination of rice (Jiang et al. 2012). The limited genetic diversity of the weedy rice accessions on the background of a simultaneously great morphological diversity might appear paradoxical, but have also been reported in other studies (Clements et al. 2004; Dlugosch and Parker 2008; Reagon et al. 2011). This gap points to a high degree of developmental plasticity, which seems to be a core element of a weedy lifestyle.

### Genetic markers reveal multiple types of weedy rice

The results of the analysis with the 19 SSR markers showed substantial genetic diversity in our weedy rice samples although the diversity varied considerably between the populations. The overall *H*_e_, as measure of genetic diversity, was 0.295 in our study. The value for H_e_ reported by Cao et al. (2006) is 0.313 is only insignificantly higher although the sampling area covered a much larger area in North-East China. The highest value (*H*_e_ = 0.478) reported so far stems from a different study in North Italy, but sampling on a much coarser-grained grid (Jiang et al. 2012). The highest degree of diversity was detected in locus RM219 (*H*_e_ = 0.640) confirming the findings of a previous study (Cao et al. 2006) on weedy rice in North-East China using the same marker set that reported a value of 0.723 for this marker. In our study, nine of the 19 loci investigated were nonpolymorphic, which explains, why the observed heterozygosity (*H*_o_) was low (Table 1). This is to be expected from the low outcrossing frequency (Jiang et al. 2012) and the fact that a relative small sampling area (130,000 ha) was sampled in high density.

The differentiation among populations can be estimated by the *F*_st_ value. The overall value for all populations in our study showed a moderate degree of differentiation (0.179). Jiang et al. (2012) found a higher degree of differentiation fitting with also the higher diversity shown by *H*_e_, which again may be due to the different design (dense sampling in our study, coarse sampling in the study by Jiang et al. (2012).

Neither Bayesian clustering (STRUCTURE) nor distance-based phylogeny (UPGMA) discriminated weedy rice and cultigens completely without transitions. The data set revealed three types of clusters mainly comprising the same individual accessions irrespective of the method used. First, clusters with exclusively weedy rice accessions; these clusters were also mostly homogenous with respect to awn morphology. Second, clusters dominated by cultigens with one or two interspersed weedy rice accessions. Third, clusters where a majority of weedy rice accessions grouped together with single cultigens. Also this third group of clusters was mostly homogenous in terms of awn morphology.

Most interesting for our question on the origin of weedy rice were the five cases, where a single cultigen was located in a cluster dominated by weedy rice accessions. Such a situation could either result from gene flow between weedy rice and cultigens, or, alternatively, point to a common ancestor. A closer look revealed that four of these five displaced cultigens with a strong affiliation to weedy rice were ancient and no longer in use today. Because they are not any longer cultivated, recent gene flow with weedy rice can be excluded as an explanation for their genetic proximity. The most straightforward model is that these cultivars are possible candidates for the ancestry of weedy rice.

Both Bayesian clustering as well as distance-based phylogeny revealed a correlation of awn morphology with the genetically defined clusters of weedy rice, most conspicuous for the mucronate accessions (Fig. 5). The fact that different awn types are maintained in an environment of mostly awnless cultivars leads to the assumption that the awn morphology might have a certain significance for weedy rice.

### Is awn morphology linked to the “weedy lifestyle”?

The current study identified several genetically defined types of weedy rice in our collection area. With exceptions of a few outliers that might indicate gene flow, these groups were defined by a relatively homogenous awn morphology. Previous studies on weedy rice have mainly focussed on hull coloration as characteristic trait. Obviously, this trait is of practical relevance, as it can be easily observed and evaluated and is also used to detect contaminations of weedy rice in commercial seeds. Nevertheless, it is still unclear, to what extent hull coloration can discriminate among populations on a genetic basis, even though previous studies have found a genetic differentiation between strawhull and blackhull populations (Gealy et al. 2002; Londo and Schaal 2007; Gealy et al. 2009; Shivrain et al. 2010; Gealy et al. 2012). As is the situation in our study, the majority of the accessions in those investigations were strawhull, and awn morphology rather than hull coloration was useful to distinguish between populations. In fact, for the awn types investigated (awnless, mucronate, black awned, straw awned), genetic clusters could be observed, where this awn type was prevalent. Thus, the analysis of awn-related traits seems to be promising to study the relationship between morphological variability and genetic structure in weedy rice.

This leads to the question on the potential biological function of awn morphology for the lifecycle of weedy rice. Awns are generally thought to promote seed dispersal via animals, because the seeds can adhere to the fur with the awn (Couvreur et al. 2004). However, whether zoochory under the conditions of intensive agriculture in North Italy is a relevant phenomenon is at least to be doubted. A second function of the awn is the regulation of seed dormancy (Gu et al. 2003, 2005). In fact, the release from seed dormancy seems to be faster in awnless populations (Fogliatto et al. 2011, 2012).

The mucronate awn type can easily be mistaken for the awnless seed types, even by the trained observer. Most contemporary rice cultivars are awnless or mucronate. This awn trait would confer to weedy rice a selective advantage over awned accessions because it would escape detection by the seed control. Additional morphological features of the mucronate group include reduced seed weight and a significantly shorter culm as compared to the other morphological groups of weedy rice. The reduced height confers a further selective advantage, because in the investigated area, weedy rice is often controlled by application of systemic herbicides with wiping bars that allow to kill the tall plants protruding from the crop canopy. The smaller stature would also help the mucronate accessions to be overlooked during manual weeding of tall rice plants, which is a further widespread practice in rice cultivation. In summary, the mucronate type of weedy rice seems to be the best adapted for a “weedy lifestyle” under conditions of this specific agricultural practice. Due to their specific morphology (awn, height), and their clear separation by genetic markers, the mucronate weedy rice populations clearly differ from the other types of weedy rice and may have their own different origin, a hypothesis that is worth to be tested by future studies.

The overall selective pressure exerted by the entire set of agronomical practices adopted in Italian rice cultivation system can be considered as relatively stable over the years and rather homogeneous across the rice cultivation area. In fact, rice is commonly cultivated as a monocrop in large parts of the Italian rice area, and more or less the same agronomical practices are applied. Under these conditions, tight mimikry of cultivated rice will increase the chance of going undetected in the field. The “mucronate strategy” should therefore lead to more stable features and a more uniform genotype as compared to the other types of weedy rice. On the contrary, awned populations which are more easily discernible and therefore under more pressure by weed control, seem to be more variable in their morphological characteristics. Their morphological variability is expected to support adaptation under conditions of changing agronomical practice. In fact, in the current study, populations with taller plants and longer seeds (traits typical in awned populations) were mainly from areas 6 and 10, where the rice cropping system had undergone significant modifications during the last years, such as the adoption of dry seeding and crop rotation.

### A look into history – old rice cultigens as ‘Trojan horses’ for weedy rice?

Rice was introduced in Italy most likely between the 13th and 15th century (Faivre-Rampant et al. 2011). Weedy rice is first mentioned by Biroli in 1807. The gap of at least 300 years between introduction of rice and emergence of weedy rice makes it unlikely that weedy rice was introduced as a contaminant with the first rice seeds imported from Asia. Since in Europe there are no wild species of *Oryza* which could be potential ancestors for weedy rice (Londo and Schaal 2007), the origin of weedy rice is most likely to be sought in the cultigens of rice themselves. But because our study lacks samples of foreign weedy rice and wild progenitors of *O. sativa,* the introduction of some weedy rice from abroad cannot completely be ruled out as a possible additional source for the emergence of weedy rice in Italy. We have therefore conducted an extensive study including Weedy accessions from Thailand and Brazil as well as of potential wild progenitor species, which will be published in a forthcoming paper. The five cultigens that turned out to be closely related to weedy rice in our study represent the most interesting candidates for possible ancestors of weedy rice. All of these cultigens detected in the weedy groups, with the exception of the recent Flipper (1997), were in use in the 19th century, when weedy rice emerged (Biroli 1807). A second factor that coincides in time is the establishment of breeding programs in Italy. Before 1800, seed material had been imported from Asia. So Bertone (1829), Ostiglia (1850) and Ranghino (1887) are direct descendants of the oldest varieties cultivated in Italy. The fact that one group of weedy rice shows great genetic similarity to a recent cultivar (Flipper) points to spontaneous mutation. In the US, a mutation from a cultivar with a white pericarp to a form with red grains was reported by Brooks et al. (2008). Similar changes could have occurred in Flipper and lead to a dedomestication resulting in new weedy rice types.

From the morphological point of view, the awn turned out to be a characteristic and important trait for weedy rice. Today, cultigens are mostly awnless. However, some of the old cultigens such as Ostiglia were still partially awned. It should be kept in mind that these old cultigens used two centuries ago were quite different from modern cultivars that are genetically homogenous and standardized, but rather resembled landraces, displaying a certain degree of genetic variability. Due to the genetic variability in these landraces a development of “weedy” traits is easily conceivable. These traits would then be positively selected under the conditions of a monocropping agriculture and then spread gradually over the agricultural area in the 200 years to follow. Until the 1960s, the practice of seeding by transplanting followed by hand weeding kept the infestation by weedy rice at a relatively low level, but with the shift to direct seeding the spread of weeds, and invasive species was promoted and weedy rice could establish in the fields.

### Conclusions and outlook

We do not think that our study has completely uncovered the enigma about the origin of weedy rice in Italy, but the results suggest at least two different origins of weedy rice in the main Italian rice area. One origin seems to be linked with the development of the first cultigens bred in Italy, the origin of the mucronate type differs and warrants further investigation. The other might be found in spontaneous mutation of recent cultivars regarding the group of weedy rice, which is genetically closely related to Flipper. This study also shows that the combination of genetic and morphological investigations can lead to results, which would not be detected when focusing only on one method. For future studies, marker sets different from ours could give more information, especially concerning the morphology. In our case, awn morphology was identified as a potential weed trait correlated with the genetic differences. However, other weed-related traits such as seed shattering ability, plant height, pericarp color, or dormancy, should be included. Furthermore, genetic marker sets connected to certain morphological traits could uncover additional aspects in the genesis of weedy rice not yet discovered in this study. In particular, the old cultivars should be investigated more carefully to understand the development of weedy populations from domesticated crops. On the other hand, weedy rice from Asia and wild rice species should be included in further studies to investigate whether additional potential sources for weedy rice types in Italy can be identified. The relatively short history of rice in Italy and its comprehensive area of cultivation provide a perfect model area for this kind of investigations. When we understand the mechanisms responsible for the genesis of weedy rice, this could also be transferred and applied to other economically important crops and their weeds. Last, but not least, we should not only see weedy rice as a weed to be contained, but as a crop wild relative weedy rice could become of interest as a genetic resource for traits useful in breeding such as the resistance to abiotic and biotic stresses, like the chilling tolerance of germination observed in Chinese accessions of weedy rice (Xia et al. 2011).
